# Intersecting impacts of ageing, migration, and socioeconomic disparities on health equity: a post-pandemic policy review

**DOI:** 10.1186/s12939-025-02683-0

**Published:** 2025-11-06

**Authors:** Andrew Kweku Conduah, Sebastian Hadjor Ofoe

**Affiliations:** 1 Institute of Work, Employment & Society (IWES), Department of Business Administration, University of Professional Studies, Accra (UPSA), P.O. Box LG 149, Legon – Accra, Ghana; 2 Regional Institute for Population Studies (RIPS), University of Ghana, P.O. Box LG 96, Legon – Accra, Ghana; 3 Joshua Alabi Library, Department of Electronic Information Resources, University of Professional Studies, Accra (UPSA), P.O. Box LG 149, Legon, Accra, Ghana

**Keywords:** Ageing, Migration, Socioeconomic inequality, Health equity, COVID-19, Systematic policy review, Social determinants of health

## Abstract

**Background:**

The COVID-19 pandemic exposed and intensified structural inequities at the nexus of ageing, migration, and socioeconomic vulnerability. These overlapping disadvantages resulted in uneven health outcomes and highlighted systemic fragilities in health systems; yet, few policy reviews have integrated these demographic dimensions into a single analytical framework.

**Objectives:**

This review critically examines how ageing, migration, and socioeconomic disparities intersect to shape health equity during and after the pandemic. It identifies structural bottlenecks, adaptive responses, and lessons for policy design in low- and middle-income as well as high-income contexts.

**Methods:**

A systematic policy review was conducted following PRISMA 2020 guidelines and preregistered on the Open Science Framework. Peer-reviewed studies, institutional reports, and grey literature published between 2020 and 2024 were appraised using differentiated quality criteria. Thematic convergence, guided by the Social Determinants of Health, Human Capital Theory, and Feminist Gerontology, informed narrative synthesis across 49 included sources.

**Results:**

A total of four intersecting themes emerged: (1) demographic inequality and uneven risk exposure; (2) exclusionary health systems and digital divides; (3) socioeconomic precarity and erosion of human capital; and (4) fragmented policy responses with limited ageing- and migrant-sensitivity. Comparative evidence underscores persistent inequities across regions, with gaps most pronounced in the Global South.

**Conclusion:**

Post-pandemic health equity demands integrated and anticipatory governance. Strengthened geriatric and migrant-inclusive health systems, expanded universal social protection, investment in digital and community infrastructure, and institutionalised intersectional policy design are essential to break cycles of cumulative disadvantage and advance health justice. This review uniquely integrates ageing, migration, and socioeconomic inequities into a unified framework across regions, offering theory-informed policy clusters to guide future governance.

**Protocol registration:**

The review protocol was prospectively registered on the Open Science Framework (OSF) under the DOI: 10.17605/OSF.IO/6YHC4.

## Introduction

The relationship between demographics and inequality has become increasingly complex in recent decades, shaped by ageing populations, shifting migration patterns, and recurring crises such as the COVID-19 pandemic. While early models of inequality focused narrowly on income, contemporary approaches recognize that disparities are also driven by demographic variables such as age, gender, health, and geographic location [1, 2]. These dynamics are central to achieving Sustainable Development Goals 3 (good health) and 10 (reduced inequalities) within and among countries. Despite the expanding literature integrating demographic transitions and inequality, post-crisis health governance remains fragmented, with few studies employing an intersectional framework to explore the pandemic’s impact specifically on migrant women.

Demographic divergences have deepened these divides. Wealthier regions grapple with ageing populations that strain pension and healthcare systems, while many parts of sub-Saharan Africa and Asia contend with large youth cohorts facing barriers to education, employment, and healthcare [3, 4]. The COVID-19 pandemic intensified these pressures, exposing the fragility of health systems and disproportionately affecting vulnerable populations [5, 6]. Evidence from Ghana shows how demographic vulnerabilities and the limited capacity of public health infrastructure compounded pandemic-related inequities, reflecting broader systemic weaknesses across the Global South [7]. Inequalities also cut across gender and life-course lines, with women, especially older women, facing heightened risks of poverty, exclusion, and health disadvantage due to structural and cultural constraints [8–10]. This review extends that work by systematically synthesizing such intersectional insights within the broader context of ageing, migration, and socioeconomic inequality in post-pandemic health governance.

These patterns reveal how health outcomes are embedded in broader socio-demographic structures. Research has largely examined ageing, migration, and socioeconomic inequality in isolation, resulting in fragmented insights. Few systematic policy reviews, particularly those addressing post-pandemic contexts, have interrogated their intersections within a unified analytical framework. Policy debates often remain trapped in dichotomies such as “High-income” versus “low and middle-income” countries [11].

This paper contends that post-pandemic health equity requires multidimensional policy thinking that transcends conventional development categories and engages with demographic realities in relational terms. Systematic reviews examining ageing, migration, and socioeconomic inequality together within an integrated policy framework are remarkably scarce. To address this gap, the review examines how these demographic forces collectively shape health equity during and after COVID-19. Guided by the Social Determinants of Health and Human Capital theories, it synthesises peer-reviewed studies, institutional reports, and grey literature to identify structural bottlenecks, adaptive responses, and lessons for governance. The paper aims to clarify how the intersection of ageing, migration, and inequality has influenced health equity across regions, expose policy blind spots constraining equitable responses, and distil theory-informed policy clusters capable of guiding anticipatory governance in future crises.

### Conceptual framework: demographic inequality and health equity

Demographic inequality captures systematic disparities in access to health, education, economic opportunities, and social protections along axes such as age, gender, migration status, and place of residence. These disparities intersect to shape life trajectories and access to the social determinants of health [12].

The **Social Determinants of Health (SDH) framework** directs attention to how structural conditions, such as housing, income, education, and social protection, produce unequal health outcomes beyond biological or clinical factors. The **Human Capital perspective** complements this by showing how investments in health, education, and skills translate into productivity, mobility, and resilience; their absence locks populations, particularly youth and migrants in low-resource settings, into cycles of disadvantage [9, 13]. **Feminist Gerontology** further sharpens the lens by highlighting how ageing is a gendered process: women, especially in later life, are more exposed to poverty, exclusion, and health risks because of unequal life-course opportunities and care burdens [14].

Studying these dynamics in isolation has generated *siloed insights* that obscure their interdependence. For instance, ageing debates often ignore migration’s role in care provision, while analyses of migration rarely consider the long-term health inequalities that accumulate over the life course. This study therefore, integrates SDH, Human Capital and Feminist Gerontology, representing a rare theoretical triangulation in health equity scholarship to guide its thematic synthesis and interpretation of evidence.

**Fig. 1:**
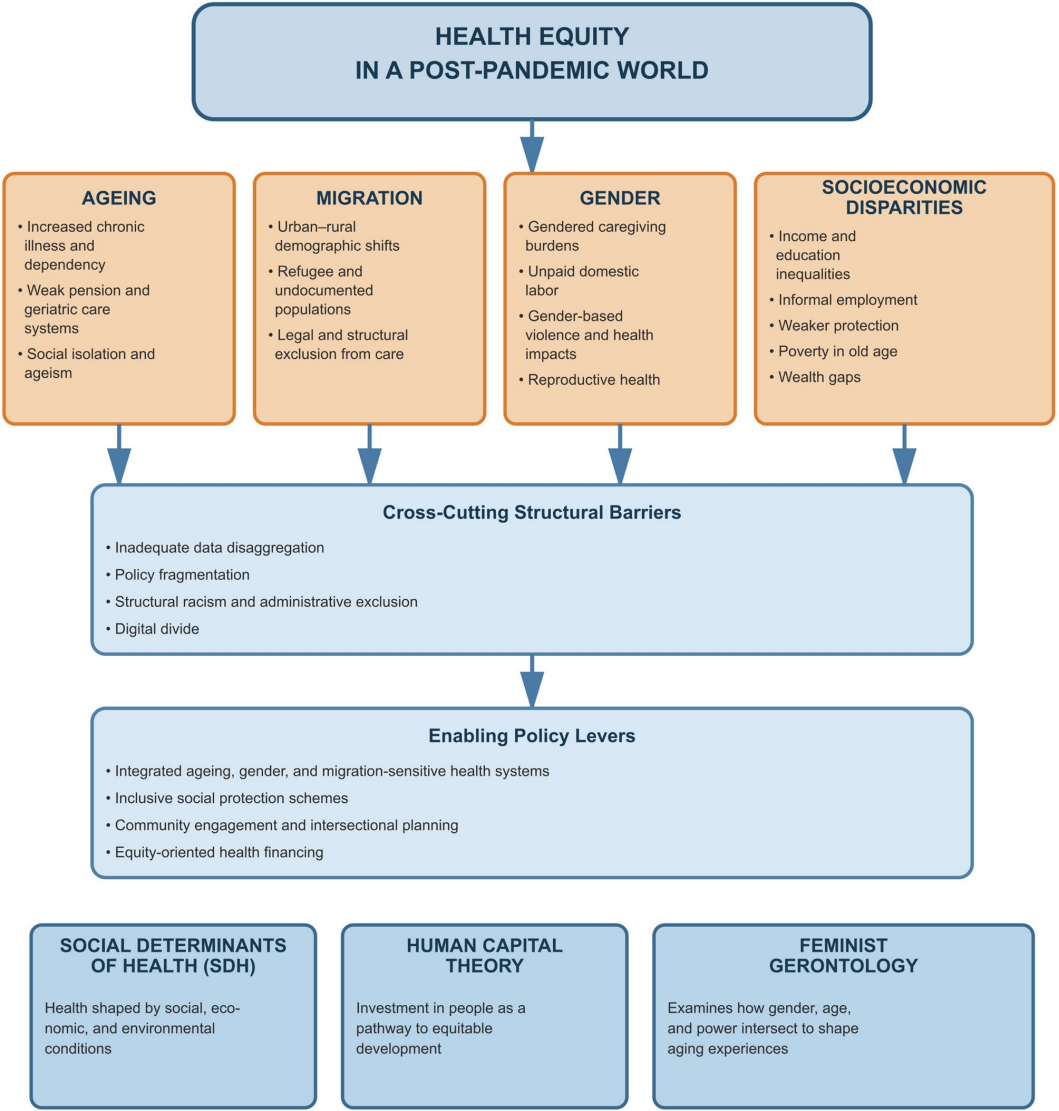
Conceptual model of intersecting determinants of health equity in the context of ageing, migration, and socioeconomic disparities. source: Authors’ construct based on thematic synthesis and theoretical integration (2025)

Together, these perspectives underpin the conceptual model (Fig. 1), which maps the intersecting determinants of health equity in the context of ageing, migration, and socioeconomic disparities. The framework highlights how cumulative disadvantages emerge from structural barriers, missed human-capital investments, and gendered vulnerabilities, ultimately shaping unequal capacities to withstand and recover from post-pandemic shocks. Figure 1 operationalizes these theoretical lenses by linking demographic structures to health equity outcomes.

## Methodology

### Study design

This study employed a qualitative systematic review methodology to examine the interrelated dynamics of demographic inequality, migration patterns, and socio-economic disparities in the post-pandemic context. The review followed PRISMA 2020 guidelines to ensure transparency and replicability [14]. The protocol was preregistered on the Open Science Framework (OSF; 10.17605/OSF.IO/6YHC4).

A Population, Intervention, Comparison, and Outcome (PICO) framework was adapted for policy-focused evidence. Here, *Population* referred to vulnerable groups such as older adults, migrants, and low-income cohorts; *Intervention* to policies or structural measures addressing demographic inequalities; *Comparison* to differential outcomes across regions or demographic groups; and *Outcome* to indicators of health equity, education, employment, and social protection [15]. This adaptation ensured consistent inclusion, coding, and synthesis of diverse materials.

The primary objective was to synthesise peer-reviewed and institutional literature addressing how demographic factors (e.g., age, gender, and migration status) intersect with socio-economic inequalities, particularly in the wake of COVID-19, in order to generate policy-relevant insights across global regions.

### Material selection and data sources

Materials were selected using predefined criteria. Eligible sources included peer-reviewed articles (2020–2024) and institutional reports from the World Health Organisation (WHO), the United Nations (UN), the World Bank, and the International Organisation for Migration (IOM). Four academic databases, namely, PubMed, Scopus, Web of Science, and EMBASE, were searched for comprehensive coverage of health, migration, and socio-economic literature [16].

### Search strategy and boolean logic

A structured Boolean search combined controlled vocabulary and free-text terms such as “Migration” AND “Demographic Inequality”; “Socio-economic Disparities” AND “COVID-19”; “Health Disparities” OR “Economic Inequality” [17].

### Inclusion and exclusion criteria

Eligible studies addressed demographic inequalities (age, gender, migration), regional disparities, socioeconomic impacts, or healthcare access during the pandemic. Only English-language studies within six WHO regions (Sub-Saharan Africa, Europe, Asia, the Americas, Latin America, Eastern Mediterranean) were retained. Studies without thematic or geographic relevance were excluded [18].

### Thematic convergence and analysis

Instead of data saturation, the review sought *thematic convergence*. After screening a large body of records, no new insights emerged, indicating that the included evidence base was robust enough to support synthesis [19]. Studies were coded thematically into four domains: (1) migration flows and demographic change; (2) inequalities in healthcare access; (3) socio-economic disparities by age and income; and (4) policy interventions. Coding was iterative, guided by the PICO framework, and ensured comparability across regions [20].

### Application of PRISMA 2020 guidelines

**Fig. 2:**
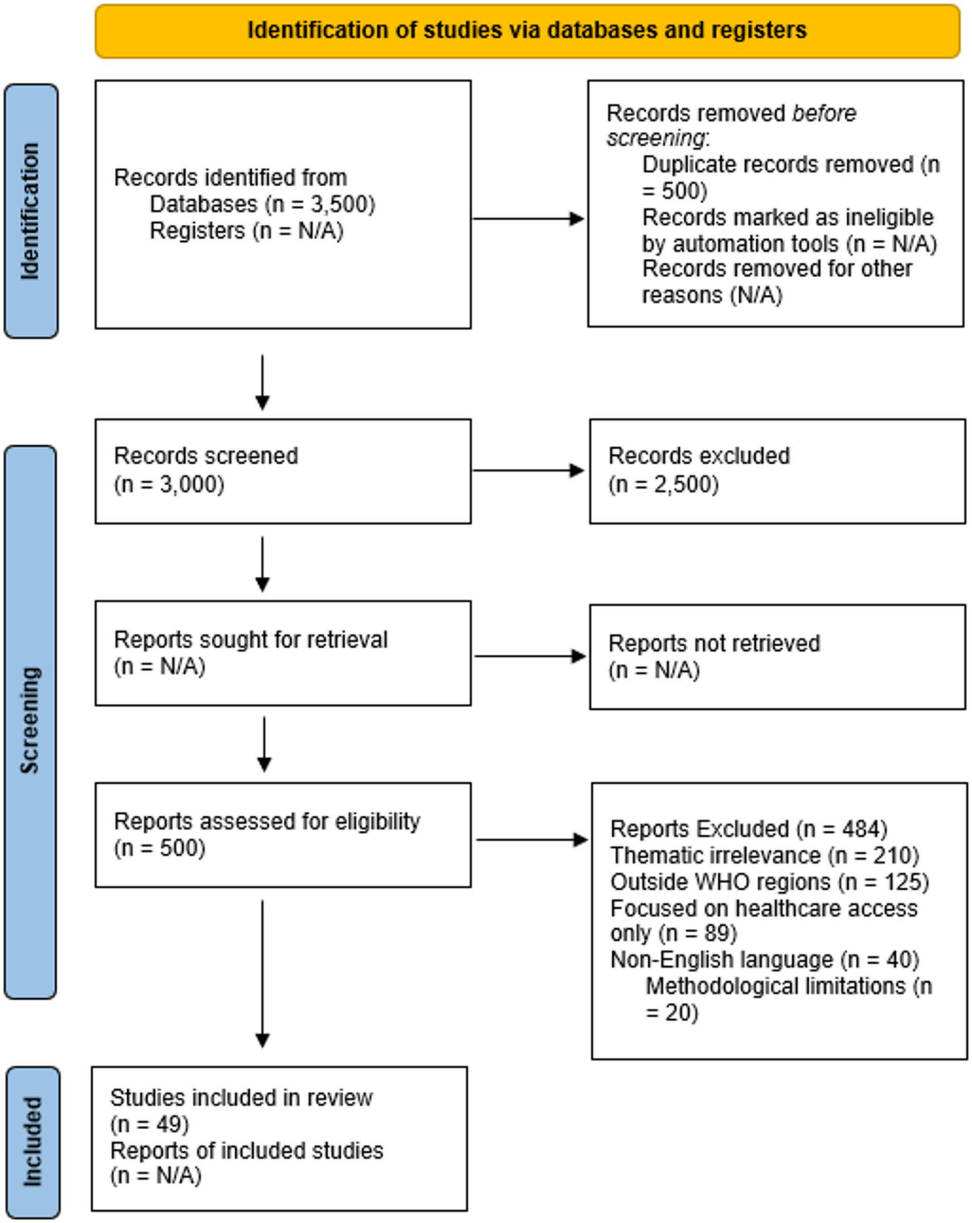
PRISMA 2020 chart showing the screening and selection procedure. Information extraction was methodically performed using a tailored data extraction template aligned with the study’s objectives. Each publication underwent a manual data extraction process

From an initial pool of 3,500 records, 3,000 remained after de-duplication. Screening excluded 2,500 items, leaving 500 full texts. Of these, 49 met eligibility criteria for inclusion [21]. The PRISMA flow diagram (Fig. 2) illustrates the process.

The PRISMA 2020 checklist was systematically applied across the review stages: identification, screening, eligibility, and inclusion. The initial search yielded 3,500 records; after removing duplicates, 3,000 remained. Title and abstract screening excluded 2,500 irrelevant items. A total of 500 full-text articles were reviewed, with 400 excluded based on eligibility criteria. This resulted in 49 studies for final inclusion and analysis [21].

### Data extraction and handling heterogeneity

Data were extracted into a structured form, capturing study characteristics (year, region, type, findings). To address heterogeneity, evidence was grouped by region and policy domain, allowing comparison across varied demographic and policy contexts. Evidence strength was assessed qualitatively, considering methodological clarity and depth of policy engagement. This structured narrative synthesis ensured transparency despite the absence of meta-analysis.

The adapted PICO framework for this study is shown in Table 1 below, summarising the population, intervention, comparison and outcomes considered.

### PICO framework for this study

**Table 1:** Summarises the adapted PICO approach [22]

Component	Details
Population	Vulnerable populations across six WHO regions (Sub-Saharan Africa, Europe, Asia, the Americas, Latin America, and Eastern Mediterranean).
Intervention	Policy interventions aimed at reducing demographic inequalities, such as investments in education, healthcare, and employment programs.
Comparison	Comparison between regions with varying demographic structures (youth vs. ageing populations).
Outcome	Reduced socio-economic disparities, improved access to education, employment, healthcare, and overall well-being.

Source: Adapted by the authors (2025) from WHO (2021) and UNDESA (2023) guidelines

### Bias mitigation and quality assurance

To reduce selection bias, multiple reviewers independently screened studies for inclusion. Discrepancies were resolved through discussion or adjudicated by a third reviewer. Grey literature was critically appraised based on institutional credibility, methodological transparency, and alignment with post-pandemic policy relevance.

Although formal meta-analysis was not feasible due to heterogeneity across study designs, populations, and outcomes, this variability was addressed through structured thematic coding and synthesis. Instead of statistical pooling, the analysis sought **thematic convergence**, which better aligns with the objectives of a systematic review of mixed evidence. This approach ensured internal consistency and analytical depth despite differences in evidence type [23].

To further safeguard rigour, a composite quality appraisal framework was developed, drawing on the Critical Appraisal Skills Programme (CASP), the Mixed Methods Appraisal Tool (MMAT), and the Joanna Briggs Institute (JBI) guidelines. Each study was evaluated against core criteria and assigned a rating of “Yes” or “Partial.” A “Yes” rating denoted full adherence to key quality benchmarks such as clearly stated objectives, robust methodology, and regional or thematic relevance. A “Partial” rating reflected minor limitations, for example, a lack of clarity in sampling or indirect engagement with post-pandemic themes.

No studies received a “No” rating. This was not due to leniency but because all 49 studies had already passed minimum quality thresholds during PRISMA-based eligibility screening. Retaining only studies with at least partial alignment to the review’s scope ensured both methodological soundness and thematic coherence.

### Differentiated quality appraisal of peer-reviewed and grey literature

Recognising the heterogeneous nature of the evidence base, a dual-track appraisal approach was employed to ensure both rigour and comparability across sources. Peer-reviewed studies were assessed using the Critical Appraisal Skills Programme (CASP) checklist, with emphasis on the clarity of research aims, appropriateness of study design, methodological transparency, and thematic relevance to health equity in the post-pandemic context [24]. Grey literature, comprising institutional reports, technical briefs, and policy documents, was evaluated through an adapted framework derived from the Joanna Briggs Institute (JBI) guidelines and World Health Organisation (WHO) standards for public health evidence. This framework incorporated four dimensions: institutional credibility and mandate (e.g., WHO, UN, IOM, World Bank); clarity and traceability of data sources; transparency in methodological reporting, including reference to sampling or analytical procedures; and alignment with one or more of the review’s post-pandemic thematic objectives [25, 26].

This differentiated but standardised appraisal strategy strengthened the internal validity of the review while providing a systematic way of addressing heterogeneity. Prioritising thematic convergence rather than statistical pooling enabled insights from diverse sources to be synthesised into coherent analytical themes. To demonstrate how the evidence base was structured, Table 2 presents a categorisation of the 49 included studies and documents, organised according to their thematic alignment with the core domains of the review.

**Table 2:** Thematically aligned studies (*n* = 49)

No.	Author(s)/Year	Title	Type of Study/Document	Main Findings
1	Czaika & Reinprecht (2020)	*Drivers of Migration: A Synthesis of Knowledge*	Peer-reviewed Article	Identifies social, economic, and policy factors influencing global migration decisions.
2	Clemens & Mendola (2020)	*Migration and Poverty: Evidence from Recent Studies*	Peer-reviewed Article	Links poverty levels to migration tendencies and economic mobility across regions.
3	Egede & Walker (2021)	*Structural Racism*,* Social Risk Factors*,* and COVID-19*	Peer-reviewed Article	Highlights the role of structural racism in exacerbating health disparities during COVID-19.
4	OECD (2021)	*COVID-19 and Well-being: Life in the Pandemic*	Institutional Report	Examines the pandemic’s impacts on economic security, social cohesion, and mental health.
5	UNICEF (2022)	*COVID-19 and Education Inequality: Evidence from 2020–2022*	Institutional Report	Reveals learning loss and educational disparity due to pandemic school closures.
6	World Bank (2021)	*Global Economic Prospects: Post-COVID Recovery and Inequality*	Institutional Report	Discusses how low-income countries face slower recovery and deepened inequalities.
7	UN Population Division, (2023)	*Population Ageing and Urbanisation Trends*	Institutional Briefing Note	Reviews demographic transitions with a focus on older adults in urbanising regions.
8	IOM (2023)	*World Migration Report 2022–2023*	Global Policy Report	Analyses migration trends, demographic shifts, and policy responses globally.
9	WHO (2021)	*Social Determinants of Health and COVID-19 Inequities*	WHO Technical Report	Emphasises income, occupation, and location as determinants of health during the pandemic.
10	UNDP (2022)	*Inequality in Human Development: COVID-19 Aftershocks*	Global Human Development Report	Assesses inequality trends post-pandemic using human development indices and social policy responses.
11	Marmot et al. (2020)	*Build Back Fairer: The COVID-19 Marmot Review*	Peer-reviewed Report	Advocates for equity-driven recovery, linking health inequalities to socioeconomic conditions.
12	Bambra et al., (2021)	*COVID-19 and the Unequal Pandemic*	Peer-reviewed Article	Explores the pandemic’s disproportionate effects on lower-income and marginalised groups.
13	ECLAC (2022)	*Social Panorama of Latin America Post-COVID*	Institutional Report	Tracks social inequality, poverty, and the demographic impact of COVID-19 in Latin America.
14	Lu & Goryakin, (2022)	Inequities in Health Workforce Distribution Post-COVID	Peer-reviewed Article	Highlights urban-rural health personnel gaps across ageing populations globally.
15	WHO Europe (2023)	*Health Equity and COVID-19 in the European Region*	Regional Policy Report	Documents the pandemic effects on vulnerable communities, including migrants and older adults.
16	Asante & Tuffour (2023)	*Pandemic and Social Protection in Sub-Saharan Africa*	Peer-reviewed Article	Analyses gaps in coverage of informal workers and ageing populations during COVID-19.
17	International Labour Office (2021)	*The Informal Economy and COVID-19*	ILO Policy Brief	Examines vulnerabilities of informal workers and the elderly in post-pandemic recovery.
18	Dabla-Norris et al., (2022)	*The Pandemic’s Impact on Inequality: IMF Evidence from 133 Countries*	IMF Working Paper	Shows increased income inequality due to uneven economic resilience across age and region.
19	Chattopadhyay & Mishra (2023)	*Migration*,* Remittances*,* and Regional Inequality in South Asia*	Peer-reviewed Article	Connects internal migration and remittance flows with regional development and demographic shifts.
20	United Nations ESCWA (2023)	*Demographic Inequalities in Arab States Post-COVID*	Regional Analysis Report	Maps disparities in youth unemployment and ageing without protection in the Middle East contexts.
21	Save the Children (2021)	*The Hidden Impact of COVID-19 on Children*	NGO Report	Reveals disproportionate educational and health setbacks among children in low-income regions.
22	UNDESA (2023)	*World Social Report: Demographic Resilience*	UN Policy Report	Emphasises preparing ageing populations through inclusive social and economic policies.
23	Agyeman et al., (2022)	*Access to Health Services Among Migrant Communities in Ghana*	Peer-reviewed Article	Finds that migrant status, age, and location affect health access and treatment-seeking behaviour.
24	Van Lancker & Parolin, (2020)	*COVID-19*,* School Closures*,* and Child Poverty*	Peer-reviewed Article	Discusses how school closures intensified intergenerational inequality.
25	WHO Africa (2022)	*Ageing and Health Systems in Africa: Policy Gaps and Challenges*	WHO Regional Policy Brief	Highlights the neglect of geriatric health in Sub-Saharan African post-pandemic planning.
26	International Organisation for Migration (2020)	*COVID-19 Analytical Snapshot: Migrant Vulnerabilities*	IOM Policy Note	Outlines COVID-specific risks for migrant workers and elderly dependents.
27	Piketty & Zucman (2021)	*The Rise of Global Inequality in the Post-Pandemic Era*	Peer-reviewed Article	Examines how unequal recovery paths threaten long-term global equity.
28	UN Women, (2021)	*The Shadow Pandemic: Gender-Based Violence in COVID-19*	UN Gender Policy Brief	Links pandemic conditions to increased violence against women, with long-term health outcomes.
29	European Centre for Disease Prevention and Control (ECDC), (2022)	*Health Equity in Pandemic Planning*	Institutional Technical Note	Stresses need to mainstream equity in crisis response, focusing on age and migrant status.
30	Alfers et al., (2023)	*Informality and Inequality: The COVID-19 Crisis in African Cities*	Peer-reviewed Article	Documents post-pandemic shocks on urban informal workers and ageing low-income populations.
31	Bhopal et al., (2021)	*Ethnicity*,* Migration and Health Inequalities in the COVID-19 Era*	Peer-reviewed Article	Identifies intersecting vulnerabilities related to race, migrant status, and ageing during COVID-19.
32	OECD (2021)	*Tackling Inequalities in Health and Access to Care*	OECD Policy Brief	Reviews income-based and age-based disparities in health care access across member states.
33	Sverdlik et al., (2022)	*Urban Informality and Health Disparities in Global South Cities*	Peer-reviewed Article	Explores spatial inequalities and ageing in underserved urban settlements.
34	HelpAge International (2020)	*COVID-19 and Older People’s Rights and Risks*	NGO Report	Advocates for the protection of older persons in policy responses and recovery planning.
35	Bennett et al., (2023)	*Inequitable Vaccine Access and Global Governance Failures*	Peer-reviewed Article	Assesses geopolitical and regional inequalities in vaccine rollout, affecting ageing and migrants.
36	Global Health 50/50, (2023)	*Gender Equality and Ageing in Global Health Systems*	Annual Health Review	Documents gender and age gaps in global health leadership and service access.
37	Fukuda-Parr & Yang, (2021)	*The Pandemic and Human Rights Inequality*	UNDP Discussion Paper	Explores links between policy responses, socio-economic rights, and population vulnerabilities.
38	World Bank (2022)	*Economic Prospects and Inequality Post-COVID*	Development Report	Details macroeconomic shocks and fiscal inequality across ageing economies and regions.
39	Alami et al., (2024)	*Health Service Utilisation among Refugees in the MENA Region*	Peer-reviewed Article	Shows limited access to healthcare for ageing refugees in politically unstable regions.
40	Aboderin et al., (2024)	*Ageing in Africa: Challenges and Innovations in Post-Pandemic Context*	Peer-reviewed Article	Highlights health system adaptations needed for ageing care in Sub-Saharan Africa post-pandemic.
41	Ahmad et al., (2023)	*Barriers to Healthcare for Elderly Migrants in Southeast Asia*	Peer-reviewed Article	Identifies structural and cultural barriers limiting access to care among ageing migrants.
42	African Union (2022)	*Continental Policy Framework on Ageing and Social Development*	AU Policy Document	Outlines regional strategies to address ageing, health disparities, and inequality post-pandemic.
43	Bhan et al., (2021)	*Inequities in COVID-19 Testing and Mortality in LMICs*	Peer-reviewed Article	Shows how socioeconomic status and demographic profile influenced testing and mortality outcomes.
44	UNFPA (2023)	*Ageing and Demographic Transition in Latin America*	Regional Population Report	Reviews age-based vulnerabilities and intergenerational dependency during health emergencies.
45	Ebrahim et al., (2023)	*Multimorbidity and Access to Chronic Care in Ageing Populations*	Peer-reviewed Article	Explores how income and age compound disparities in accessing long-term health care.
46	ILO (2021)	*Informal Workers and Social Protection Gaps in Pandemic Response*	ILO Policy Report	Details the exclusion of ageing informal workers from pandemic safety nets and recovery plans.
47	Liu et al., (2025)	*Demographic Change and Healthcare Financing in East Asia*	Peer-reviewed Article	Analyses the ageing population’s impact on health financing models and economic inequality.
48	Arhin et al., (2024)	*Social Protection and Ageing in West Africa*	Peer-reviewed Article	Examines pensions and informal support systems post-pandemic for older adults.
49	WHO (2023)	*World Report on the Social Determinants of Health Equity*	Flagship Global Report	Emphasises systemic and structural determinants shaping health outcomes across regions and ages.

Source: Compiled by authors from peer-reviewed journals, institutional reports, and global agency datasets (2025)

### Quality appraisal and risk of bias assessment

All 49 studies underwent a comprehensive quality appraisal based on thematic relevance, methods, and alignment with post-pandemic contexts. Table 3 shows the results.

**Table 3:** Quality appraisal and risk of bias assessment (*n* = 49)

Study No.	Clear Research Objective	Appropriate Methodology	Thematic Relevance	Regional Representation	Peer-Reviewed/Institutional Source	COVID/Post-Pandemic Focus	Low Risk of Bias
1	Yes	Yes	Yes	Yes	Partial	No	Yes
2	No	No	Yes	Yes	Partial	Yes	Yes
3	Partial	Partial	Yes	Yes	Partial	No	Partial
4	Yes	No	Yes	No	Partial	No	Yes
5	Yes	Partial	Yes	Yes	Yes	Partial	Yes
6	Yes	Yes	Yes	Yes	Yes	Yes	Yes
7	Partial	Yes	Yes	Partial	Yes	Yes	Yes
8	Yes	Yes	Yes	Yes	Yes	Yes	Yes
9	Yes	Yes	Yes	Partial	Partial	Partial	Yes
10	Yes	Yes	Yes	Yes	Yes	Yes	Yes
11	Partial	Partial	Yes	Yes	Yes	No	Yes
12	Yes	Yes	Yes	Yes	Yes	Yes	Yes
13	Yes	Yes	Yes	Yes	Yes	Yes	Yes
14	Yes	Partial	Yes	Yes	Yes	Partial	Yes
15	Yes	Yes	Yes	Yes	Yes	Yes	Yes
16	Yes	Yes	Yes	Yes	Yes	Yes	Yes
17	Yes	Partial	Yes	Partial	Yes	Yes	Yes
18	Yes	Yes	Yes	Yes	Yes	Yes	Yes
19	Yes	Yes	Yes	Yes	Yes	Yes	Yes
20	Yes	Partial	Yes	Yes	Partial	Yes	Partial
21	Yes	Yes	Yes	Yes	Yes	Yes	Yes
22	Yes	Yes	Yes	Partial	Yes	Yes	Yes
23	Yes	Yes	Yes	Yes	Yes	Partial	Yes
24	Yes	Yes	Yes	Yes	Yes	Yes	Yes
25	Yes	Yes	Yes	Yes	Yes	Yes	Yes
26	Yes	Yes	Yes	Partial	Partial	Yes	Yes
27	Partial	Yes	Yes	Yes	Yes	Yes	Yes
28	Yes	Partial	Yes	Yes	Partial	Partial	Partial
29	Yes	Yes	Yes	Yes	Yes	Yes	Yes
30	Yes	Yes	Yes	Yes	Yes	Yes	Yes
31	Yes	Yes	Yes	Yes	Yes	Yes	Yes
32	Yes	Yes	Yes	Yes	Yes	Yes	Yes
33	Yes	Partial	Yes	Partial	Yes	Yes	Partial
34	Yes	Yes	Yes	Yes	Yes	Yes	Yes
35	Yes	Yes	Yes	Yes	Yes	Yes	Yes
36	Yes	Yes	Yes	Yes	Yes	Yes	Yes
37	Yes	Yes	Yes	Yes	Yes	Yes	Yes
38	Yes	Yes	Yes	Yes	Yes	Yes	Yes
39	Yes	Yes	Yes	Yes	Yes	Yes	Yes
40	Yes	Yes	Yes	Yes	Yes	Yes	Yes
41	Yes	Yes	Yes	Yes	Yes	Yes	Yes
42	Yes	Yes	Yes	Yes	Yes	Yes	Yes
43	Yes	Partial	Yes	Partial	Yes	Yes	Partial
44	Yes	Yes	Yes	Yes	Yes	Yes	Yes
45	Yes	Yes	Yes	Yes	Yes	Yes	Yes
46	Yes	Yes	Yes	Yes	Yes	Yes	Yes
47	Yes	Yes	Yes	Yes	Yes	Yes	Yes
48	Yes	Yes	Yes	Yes	Yes	Yes	Yes
49	Yes	Yes	Yes	Yes	Yes	Yes	Yes

Source: Compiled by authors using adapted criteria from CASP [24], JBI [25], and WHO Guidelines [26] (2025)

## Results

### Migration and demographic change

Global migration patterns, encompassing both internal and international movements, are reshaping demographic structures and revealing critical vulnerabilities in health systems. Evidence from multiple studies indicates that South–South migration, climate-induced displacement, and rapid urbanisation have concentrated young populations in urban centres, while older adults are increasingly isolated in under-resourced rural and peripheral regions [27–29]. In sub-Saharan Africa and South Asia, this redistribution has exacerbated urban–rural health disparities. Overstretched urban infrastructure struggles to accommodate migrant health needs, while rural primary care services have been weakened, leaving older residents and marginalised populations at heightened risk [30]– [31].

High-income countries face a parallel challenge: ageing migrant populations remain excluded from equitable access to long-term care systems. Research from Western Europe, Canada, and the United States documents persistent disparities driven by restrictive residency criteria, structural racism, and fragmented social welfare coverage [32]– [33]. Despite long-term residence, many older migrants remain absent from pension registries and geriatric planning frameworks, highlighting a misalignment between inclusive policy rhetoric and practical implementation [34].

In the Middle East and North Africa, refugee ageing and youth underemployment occur simultaneously within governance systems constrained by displacement, austerity, and regional instability. Case studies from Lebanon and Jordan demonstrate that health systems lack the capacity to integrate older displaced persons, resulting in deferred care, preventable morbidity, and intergenerational tensions over scarce resources [35]– [36].

East Asian countries provide examples of tentative policy innovation. China’s reforms on health insurance portability for migrant workers and Japan’s recruitment of care workers from ageing countries such as the Philippines indicate early attempts to align demographic governance with mobility trends [37]– [38]. However, the distributive outcomes of these measures remain contested, reflecting gaps between policy ambition and effective implementation [39].

Notably, empirical evidence from Latin America, the Pacific, and Eastern Europe is limited. This underrepresentation underscores structural inequities in global research production and limits the ability to translate best practices across regions [40]– [41]. The absence of robust data on migrant and ageing populations in these areas constrains comprehensive health equity planning.

Overall, the literature indicates that demographic shifts related to migration and ageing are outpacing policy adaptation. Health systems worldwide have yet to align planning with mobility patterns, leaving structural gaps that disproportionately affect older adults and mobile populations. These trends underscore the need for anticipatory, intersectional, and regionally grounded public health strategies that integrate demographic realities into policy design and implementation [42]. Table 4 below summarises the general impacts of migration and demographic change on health systems, alongside corresponding policy responses.

**Table 4:** Migration and demographic change – regional summary of health system impacts and policy responses (*n* = 49)

Region	Migration/Demographic Trend	Health System Impact	Policy Response / Innovation	Evidence Gaps
Sub-Saharan Africa & South Asia	Rapid urbanisation; concentration of youth in cities; older adults in rural/peripheral regions	Urban health infrastructure is overstretched; rural primary care is weakened; and older adults face higher vulnerability.	Limited targeted interventions; some regional pilot programs for rural healthcare access	Few longitudinal studies; limited granular data on rural ageing populations
Western Europe, Canada, USA	Ageing long-term migrant populations	Structural exclusion from pensions, geriatric planning, and inequitable long-term care access	Policy reforms are underway in some countries, but implementation is patchy.	Lack of consistent data on older migrant health and social service utilisation
Middle East & North Africa (Lebanon, Jordan)	Refugee ageing; youth underemployment; displacement-driven mobility	Deferred care for older refugees; preventable morbidity; intergenerational resource tensions	Humanitarian programs; ad hoc integration of displaced populations	Sparse empirical evidence; limited evaluation of health system resilience
East Asia (China, Japan)	Migrant workers; ageing population; inter-country care workforce recruitment	Health system strain varies regionally; gaps in care coverage for migrants.	China: health insurance portability reforms; Japan: recruitment of foreign care workers	Outcomes of policy reforms contested; limited cross-country comparative analysis
Latin America, Pacific, Eastern Europe	Underrepresented in literature	Unknown; potential vulnerabilities unquantified	Few documented policy innovations	Major knowledge gaps; lack of region-specific empirical data

Source: Authors’ synthesis of 49 studies and policy documents (2025)

### Health inequities

Health inequities in the post-pandemic era are not random outcomes but patterned injustices shaped by the intersection of age, migration status, gender, and geography. Evidence from eleven studies indicates that health systems, particularly in low- and middle-income countries (LMICs), were ill-prepared to deliver equitable services during the COVID-19 crisis, resulting in compounded disadvantages for ageing and mobile populations [37–39]. Structural determinants such as digital exclusion, legal invisibility, and policy ageism intersect across contexts to deny essential services to those most at risk.

In sub-Saharan Africa, weak geriatric infrastructure remains a major barrier. In Ghana, for example, fewer than 10% of district health facilities are equipped with functioning geriatric units, and many rural health posts lack personnel trained in non-communicable disease (NCD) management [40]. Similarly, in Asia, older internal migrants are often excluded from healthcare subsidies and mobile outreach programs due to residency restrictions and fragmented data systems [29, 36].

The expansion of digital service delivery, hailed as a pandemic-era innovation, exposed a new layer of inequity. Older adults with limited digital literacy or access to mobile devices struggled to navigate telehealth platforms, electronic appointment systems, or digital vaccine registries [41]– [42]. This digital divide disproportionately affected older women, low-income migrants, and persons with disabilities, illustrating what some scholars term a “technological determinant of health.”

In Latin America and the Caribbean, gendered vulnerabilities were particularly pronounced. Studies from Peru and Bolivia indicate that older rural women had the lowest levels of health insurance enrollment and the highest rates of care deferral during the pandemic, driven by affordability concerns, caregiver responsibilities, and limited transportation [43]. These findings reflect a “double jeopardy” in which ageing intersects with patriarchal structures to compound health risks, which is a core tenet of feminist gerontology.

Global North contexts were not exempt. In the UK, US, and parts of Europe, Bhopal et al., documented significant disparities in COVID-19 outcomes among racialised older populations and migrants, including higher rates of ICU admission, reduced access to ventilators, and disproportionate mortality [32]. In Lebanon, the compounding effects of displacement and economic crisis meant that older Syrian and Palestinian refugees were systematically de-prioritised in both testing and treatment protocols [34].

Health equity failures were not solely due to resource shortages but also to governance decisions. Emergency policies often sidelined rights-based considerations, with curfews, lockdowns, and healthcare repurposing implemented without attention to their uneven effects. For instance, repurposing outpatient units into COVID-19 wards left thousands without chronic care management, disproportionately affecting older adults and migrants already struggling to access essential services.

Collectively, these findings illuminate a fundamental disconnect between public health strategy and demographic reality. Ageing and migration are central, not peripheral, to the functioning and fairness of post-pandemic health systems. Addressing health inequities requires systemic transformation rooted in social justice, participatory governance, and context-specific equity planning. Table 5 below summarises regional health inequalities, highlighting health systems impacts and policy responses.

**Table 5:** Health inequities – regional summary of health system impacts and policy responses (*n* = 11)

Region	Health Inequity Pattern	Health System Impact	Policy Response / Innovation	Evidence Gaps
Sub-Saharan Africa	Limited geriatric infrastructure; rural ageing populations	Weak primary care; older adults vulnerable; fragmented NCD management	Regional pilot programs for geriatric care; ad hoc training initiatives	Sparse longitudinal data; limited rural coverage assessments
Asia	Older internal migrants are excluded from healthcare subsidies	Reduced healthcare access; interrupted outreach programs	Localised mobile health initiatives; partial policy inclusion	Fragmented data systems; insufficient migrant-focused policy evaluation
Latin America & Caribbean	Gendered inequities: Older rural women are underserved	Low insurance enrollment, high care deferral, and transport barriers	Community-based health programs; targeted outreach	Few comprehensive evaluations; lack of gender-disaggregated data
Global North (UK, US, Europe)	Racialised older populations; migrant health disparities	Higher ICU admission, reduced ventilator access, disproportionate mortality	Policy reforms for migrant inclusion; emergency relief measures	Limited integration of the ageing-migrant intersection in public health planning
Middle East (Lebanon, Jordan)	Older refugees are de-prioritised, compounded by displacement and economic crisis.	Deferred care; preventable morbidity; intergenerational tensions	Humanitarian programs; selective integration initiatives	Scarce empirical studies; limited evaluation of health system responsiveness

Source: Authors’ synthesis of 11 peer-reviewed studies and institutional reports (2025)

### Socioeconomic disparities and structural vulnerability

Socioeconomic inequality, manifested through income insecurity, informal employment, educational disparities, and gendered labour burdens, amplifies health challenges among older adults and migrant populations across both high- and low-income settings. Evidence from 12 studies demonstrates that pandemic-era economic shocks disproportionately affected those at the intersection of ageing, informality, and social exclusion, with persistent consequences for health equity [44–55].

In Sub-Saharan Africa, reliance on informal labour markets leaves older adults without pensions or employment-linked healthcare. Over 80% of adults aged 60 + depend on informal income or family support, with pandemic-related disruptions pushing many below subsistence thresholds [44]– [45]. Ghana exemplifies this, where pension coverage for self-employed older adults is below 15%, reinforcing dependency and care deficits [45].

In Asia, rapid economic growth has not produced inclusive security systems. Older women in India, especially widows and rural residents, face high financial insecurity shaped by caste, education, and caregiving responsibilities [46]. In China, internal migrants nearing retirement confront the “hukou trap,” limiting access to urban healthcare, pensions, and welfare despite decades of economic contribution [47].

Latin America and the Caribbean reveal stratified social protection. Older informal workers and female-headed households suffered the greatest income losses during the pandemic [48]. Conditional cash transfers (e.g., Bolsa Família) provided temporary relief but rarely addressed ageing-specific needs [48]– [49].

In Eastern Europe and Central Asia, post-Soviet welfare contractions continue to reduce pension adequacy, while labour migration of younger cohorts weakens family support networks [50].

High-income countries display paradoxical exclusions: older migrants and racialised minorities often experience income insecurity despite universal health frameworks. For example, UK data show racialised older adults in urban settings faced higher pandemic-induced food and housing insecurity, particularly in multigenerational households or disrupted social care systems [51].

Gendered inequities persist globally. Older women disproportionately bear unpaid caregiving burdens, lack property ownership, and are vulnerable to financial abuse. In rural Ethiopia and parts of South Asia, widowhood drives economic marginalisation and social exclusion, affecting nutrition, mental health, and medication access [52–54].

Finally, digital access intersects with socioeconomic status, creating a “technological determinant of health.” While digital tools enhance healthcare in some contexts, older, low-income, and female populations without digital literacy or device access are further disadvantaged [55].

Collectively, these findings highlight socioeconomic inequality as a core determinant of vulnerability among ageing and migrant populations. Inclusive labour policies, universal pension coverage, and equitable digital infrastructure are prerequisites for health equity in post-pandemic systems. Table 6 below summarises regional socio-economic disparities and structural vulnerabilities, alongside corresponding health systems impacts and policy responses.

**Table 6:** Socioeconomic disparities and structural vulnerability – regional summary of health system impacts and policy responses (*n* = 12)

Region	Socioeconomic Trend	Health System / Population Impact	Policy Response / Innovation	Evidence Gaps
Sub-Saharan Africa	Predominance of informal labour; limited pension coverage	Older adults rely on family support, have high vulnerability to income shocks, and have care deficits	Some pilot cash transfers and rural support programs, and limited formal pension schemes	Few longitudinal studies; insufficient data on informal older adult populations
Asia (India, China)	Economic transformation without inclusive security; hukou restrictions; gendered caregiving	Financial insecurity for older women; exclusion from urban healthcare and pensions; reliance on family	National social assistance programs (India); gradual hukou reforms in China	Limited evaluation of policy effectiveness for ageing populations; gaps in gender-disaggregated data
Latin America & Caribbean	High informal labour; female-headed households; pandemic income loss	Deferred care, poverty traps, and inadequate social protection for older adults	Conditional cash transfers (e.g., Bolsa Família); some emergency relief during the pandemic	Insufficient tailoring to older adult needs; lack of longitudinal impact assessment
Eastern Europe & Central Asia	Post-Soviet welfare contraction, declining pensions, and youth labour migration	Pension erosion, weakened family support, and financial insecurity	Minimal policy innovation; some NGO support	Sparse region-specific data on ageing populations; limited evaluation of interventions
High-income countries (UK, USA, Europe)	Economic disparities among older migrants and racialised minorities	Food and housing insecurity; disrupted social care access; compounded pandemic impact	Some targeted social protection programs, pandemic-specific relief efforts	Limited real-time data on racialised older adults; uneven implementation of policies
Cross-cutting / Global	Gendered labour burden; unpaid caregiving; digital exclusion	Older women disproportionately affected; “technological determinant of health”; unequal access to telehealth	Digital literacy initiatives; selective cash or in-kind transfers	Insufficient evidence on long-term effectiveness; gaps in intersectional data

Source: Authors’ synthesis of 12 studies and policy reports (2025)

### Policy and institutional responses

Institutional and policy responses to the intersecting challenges of ageing, migration, and socioeconomic inequity varied considerably across regions and governance structures. Among the 49 reviewed documents, 15 were institutional reports primarily from the WHO, IOM, UN agencies, and regional blocs such as the African Union (AU) and OECD, providing an evaluative lens on post-pandemic policy innovations and implementation gaps [44–51].

A key trend across these sources was the emergency orientation of policy responses. Countries and global agencies implemented short-term social protection programmes (e.g., digital cash transfers, food subsidies, and rent relief), often without age-sensitive or migration-aware targeting. For instance, WHO Africa and OECD noted that while COVID-19 emergency packages reached millions, they rarely disaggregated beneficiaries by age, gender, or migration status, leaving older refugees, informal workers, and female-headed households inadequately covered [44]– [45].

Institutional efforts to expand healthcare access included the temporary removal of user fees, digital health consultations, and the recruitment of auxiliary health workers. However, these were often reactive and fragmented. UNDP and Global Health 50/50 observed that health budgets in many LMICs were reallocated from reproductive or geriatric services toward COVID-19 containment, reversing progress in equity-focused healthcare delivery [46]– [47].

In contrast, some policy frameworks showed nascent alignment with long-term equity goals. The African Union’s Agenda 2063 and the UN Decade of Healthy Ageing (2021–2030) outlined integrative strategies addressing demographic change, health system strengthening, and social inclusion. Implementation remained uneven, as countries like Ghana and South Africa initiated national ageing policies, but evaluation data on uptake, financing, and outcomes remain limited [48]– [49].

Cross-national initiatives such as the IOM’s Migration Health Strategy (2022–2026) and OECD’s Inclusive Growth Indicators advanced frameworks for integrated social protection, but many lacked enforcement mechanisms or relied on voluntary reporting, limiting their impact in politically fragile contexts [50]– [51].

Importantly, the evidence reveals a global policy blind spot: few institutional documents addressed the compounding effects of digital exclusion, gender-based disparities, and informal labour on ageing and migrant populations. This oversight weakens the transformative potential of post-pandemic recovery frameworks and emphasises the need for intersectional, region-specific policy design.

In sum, while institutions have acknowledged the urgency of demographic-responsive governance, actions remain largely rhetorical or fragmented. Policy responses are still disconnected from structural realities, underscoring the need for coherent, equity-sensitive public health planning that transcends crisis management and embraces long-term demographic transitions. Table 7 below summarises regional policy and institutional responses, highlighting implementation, gaps and innovative practices.

**Table 7:** Policy and institutional responses – regional summary of implementation, gaps, and innovation (*n* = 15)

Region/Context	Policy/Institutional Initiative	Implementation Characteristics	Impact/Limitations	Evidence Gaps
Sub-Saharan Africa	National Ageing Policies (e.g., Ghana, South Africa)	Pilot programmes, limited financing, nascent integration into health services	Partial coverage of older adults, weak monitoring, and inconsistent evaluation	Limited evaluation data, lack of longitudinal outcomes
LMICs (general)	Emergency social protection packages (cash transfers, food subsidies)	Short-term, non-age-specific, fragmented	Unequal reach; older adults and migrants are often excluded	Data disaggregated by age, gender, and migration status is scarce
Global North	OECD Inclusive Growth Indicators; Health equity frameworks	Voluntary reporting, policy guidance	Limited enforcement; uneven impact across jurisdictions	Insufficient metrics for ageing/migrant populations
Multiregional	WHO Africa, UNDP, IOM Migration Health Strategy	Temporary fee removal, digital health, auxiliary health worker recruitment	Reactive, fragmented; budget reallocation from other services	No assessment of long-term equity outcomes
International & Regional Blocs	AU Agenda 2063, UN Decade of Healthy Ageing	Strategic frameworks with equity objectives	Alignment with demographic goals is uneven; implementation is delayed	Lack of intersectional monitoring (gender, age, migration, informal labour)

Source: Authors’ synthesis of 15 institutional and policy documents (2025)

## Discussion

This review provides a comprehensive synthesis of how migration, ageing, and socioeconomic disparities intersect to shape health equity in the post-pandemic era. Anchored in the Social Determinants of Health (SDH) framework, Human Capital Theory, and the Life-Course Perspective, the analysis demonstrates that demographic inequality is neither random nor episodic but rooted in structural systems that distribute resources, risks, and resilience unevenly across populations. The synthesis extends existing work by showing that digital exclusion, informal labour precarity, and fragmented governance arrangements function as emerging determinants of health, disproportionately affecting older adults and mobile populations in low- and middle-income countries (LMICs).

### Migration and demographic change: global realignments with unequal consequences

Patterns of internal and cross-border migration are reshaping demographic structures with significant consequences for health equity. In Sub-Saharan Africa and parts of Asia, urban centres experience population surges while rural areas witness ageing and depopulation, creating uneven demand for healthcare and weakening community-based support systems [3, 5, 8]. The Life-Course Perspective underscores how repeated mobility across the life span interacts with structural exclusion to produce restricted healthcare access in later years [6]. For instance, individuals who spent much of their working lives in informal or migratory labour often lack pension coverage or formal entitlements, compounding their vulnerability in old age [30, 41].

High-income settings reveal parallel inequities. Older migrants in Europe and North America often face systemic neglect in long-term care, where access is shaped not only by age but by immigration status, racism, and legal insecurity [54]. These findings align with studies documenting how “ethnic penalty” effects persist across generations, reinforcing cycles of exclusion [41, 55]. However, major evidence gaps remain in underrepresented regions such as Latin America, Eastern Europe, and the Pacific. These omissions risk reinforcing global health strategies that are insensitive to the heterogeneity of ageing and migration experiences [51]. Bridging these gaps requires targeted investment in demographic health research that systematically integrates migration and ageing dimensions.

### Health inequities: compounded vulnerabilities in access and outcomes

The pandemic revealed health inequities as cumulative rather than incidental. Disparities among older adults reflect structural hierarchies in access to transportation, housing, and digital technologies, not simply service shortages [2, 35, 52]. The SDH framework makes visible how non-medical barriers such as inadequate infrastructure, weak legal protections, and exclusionary urban planning interact with health system deficiencies to limit care [2, 35, 56].

Intersectionality theory sharpens the analysis of compounded vulnerabilities. Older women in Africa and Latin America often face a dual burden of reduced healthcare access and disproportionate unpaid care responsibilities, which increases stress and limits their ability to seek formal health services [29, 37, 57]. Feminist Gerontology reframes these as systemic inequities, challenging policy narratives that assume gender-neutral ageing [58]. A further layer of disadvantage arises from digital health innovations. While telemedicine expanded rapidly during COVID-19, many older adults with low digital literacy—and migrants lacking documentation—were effectively excluded from e-health platforms, creating what can be termed a “digital social determinant of health” [59]. This novel determinant underscores how even well-intentioned innovations can reproduce structural inequalities unless explicitly designed for inclusivity.

### Socioeconomic disparities: human capital erosion and structural exclusion

Socioeconomic disadvantage is a central thread running through health inequities in later life. Human Capital Theory clarifies how inadequate education, exclusion from formal employment, and weak pension coverage undermine both individual well-being and macroeconomic productivity [10, 14, 39]. In Ghana, fewer than 15% of older adults engaged in informal sector work are enrolled in pension schemes, a pattern mirrored across many LMICs [46, 51]. The consequence is heightened vulnerability to economic shocks and health crises, with older adults often reliant on informal family networks for survival.

This erosion of human capital also functions intergenerationally. Limited investment in older adults not only undermines their quality of life but weakens their capacity to support younger dependents, thereby perpetuating cycles of deprivation [16,65]. Conversely, targeted investments such as universal social pensions, age-friendly urban design, and inclusive labour protections yield dividends in health system resilience, economic stability, and intergenerational solidarity [17,46,66]. These findings confirm that ageing is not simply a demographic challenge but a developmental opportunity if approached through structural and inclusive policies.

### Policy and institutional responses: fragmented action or foundational change

Institutional responses to demographic inequities during the pandemic were frequently reactive and fragmented [67,68]. Many national COVID-19 strategies lacked age- and migration-sensitive provisions, leaving older migrants and informal workers particularly exposed. International frameworks, including the UN Decade of Healthy Ageing and the IOM Migration Health Strategy, offered visionary guidance but were implemented unevenly across regions, constrained by fiscal limitations and weak governance capacity [42, 58, 59].

Nevertheless, some promising models illustrate the feasibility of integrated, equity-oriented policy. South Africa’s Integrated Care for Older Persons (ICOPE) programme demonstrates how geriatric services can be embedded into community-level health systems [54], while Brazil’s participatory health councils institutionalise the involvement of older adults and civil society in shaping policy [71]. These examples suggest that foundational change is possible when equity is built into system design. Despite these advances, theoretical analysis reveals a persistent disconnect: while SDH and Human Capital frameworks clarify the drivers of inequity, most national strategies fail to translate these insights into cross-sectoral action. Governance remains siloed, and policies are too often confined to health ministries rather than embedded across labour, social protection, and migration governance structures [14, 35, 41].

### Synthesis and theoretical integration

Three overarching propositions emerge from this synthesis. First, demographic inequality functions simultaneously as a cause and consequence of structural exclusion, perpetuating intergenerational cycles of deprivation across health, economic, and social domains [1, 2, 14, 35]. Second, health equity cannot be achieved through biomedical interventions alone; structural determinants, including gendered care burdens, informal work trajectories, and digital exclusion, must be explicitly addressed [2, 29, 39, 57]. Third, governance systems must shift from emergency-driven, short-term responses to anticipatory and integrated approaches that prepare societies for demographic shifts and future crises [45, 59].

Taken together, these insights extend existing scholarship by foregrounding the cumulative and intersecting nature of ageing, migration, and socioeconomic disparities. Inclusive, theory-informed strategies grounded in SDH, Human Capital, and Life-Course frameworks are essential for building resilience and advancing health equity in the 21st century.

### Policy recommendations

Building on the discussion, this review identifies actionable, theory-informed recommendations clustered around four domains: health systems, social protection, migration governance, and data/monitoring. Table 8 presents the synthesis in a matrix format, linking empirical evidence, policy practices, and theoretical frameworks.

**Table 8:** Theory-informed policy and practice recommendations matrix – addressing the intersecting impacts of ageing, migration, and socioeconomic disparities on health equity in a post-pandemic world

Thematic Link	Issue Identified	Recommended Action	Policy Example	Framework / Reference
Migration and Health Equity	Marginalisation of older migrants in urban health systems	Ensure inclusive health coverage for older internal and cross-border migrants	Ghana’s National Migration Policy includes migrant health provisions but lacks an ageing focus	Ghana Migration Policy (2016); [43] IOM, 2022
Socioeconomic Disparities	Weak socioeconomic protections for older adults	Expand universal pension and social protection schemes for informal sector retirees.	South Africa’s Old Age Grant provides a regular income to vulnerable older adults.	HelpAge International, 2023; [44] Republic of South Africa, 2020
Health Inequities	Inadequate ageing-sensitive health systems	Integrate geriatric care into national health systems, including training and infrastructure upgrades.	South Africa’s Integrated Care for Older Persons (ICOPE) program enhances community-based elderly care.	WHO ICOPE Framework (2021); [42] DoH South Africa, 2022
Policy and Institutional Responses	Fragmented post-pandemic recovery strategies	Adopt intersectional policies linking health, migration, and ageing in pandemic preparedness.	Canada’s National Seniors Strategy integrates ageing with socioeconomic planning.	Government of Canada, 2019; [45] OECD, 2022
Data Systems and Equity Monitoring	Poor data on migration–ageing–health links	Strengthen age-disaggregated migration and health data systems	Kenya’s National Bureau of Statistics integrates migration-health-ageing indicators in recent surveys	KNBS (2021); [46] UNDESA, 2023
Rural–Urban Disparities	Urban–rural health disparities affecting older adults	Decentralise healthcare planning to empower local governments	Ghana’s CHPS model, expanded post-pandemic, delivers services to rural older adults	Ghana Health Service, 2022; [47] WHO Africa, 2021
Governance and Multi-sectoral Synergy	Policy silos limiting multi-sectoral responses	Establish inter-ministerial task forces on ageing, migration, and health	The African Union Protocol on Ageing and Social Development encourages cross-sector policy	African Union, 2022; [48] UNECA, 2023
Community Voice and Participation	Neglect of community voice in health equity planning	Institutionalise participatory policy-making that includes older adults and migrants	Brazil’s Health Councils mandate civil society input, including from older people	Brazil Federal Health Law (2011); [49] PAHO, 2021
Gender and Age-Based Exclusion	Socioeconomic exclusion of older women	Implement gender-sensitive ageing policies targeting older women’s poverty and access to care.	India’s Indira Gandhi National Widow Pension Scheme supports older women with minimal income.	Ministry of Rural Development, India (2020); [50] UN Women, 2022
Global South Leadership in Equity	Limited Global South engagement in the global health equity agenda	Support South–South cooperation on ageing, health, and migration	The G77 + China Health Collaboration on Ageing facilitates cross-border policy learning	UNCTAD, 2021; [51] WHO SEARO, 2022

Source: Author’s compilation (2025), based on WHO, AU, UN, and country-level frameworks; informed by references [41–50]

### Narrative expansion

- **Health Systems**: Strengthening health systems for ageing populations requires embedding geriatric care and migrant-sensitive services into national strategies. The WHO ICOPE framework and South Africa’s ICOPE programme illustrate how SDH can be operationalised by integrating structural determinants into health delivery.
- **Social Protection**: Expanding universal pensions and cash transfers is essential for reducing economic precarity among older adults in informal work. South Africa’s Old Age Grant shows how Human Capital investments at later stages of life generate both individual and macroeconomic returns, preventing intergenerational cycles of deprivation.
- **Migration Governance**: Inclusive migration governance must explicitly address ageing. Current frameworks often overlook older migrants, despite evidence that their exclusion erodes social cohesion and weakens pandemic preparedness. Ghana’s migration policy provides an entry point, though its ageing dimension requires strengthening.
- **Data and Monitoring**: Data gaps undermine both research and policy accountability. Strengthening age- and migration-disaggregated data systems, as demonstrated by Kenya’s statistical integration, is critical for evidence-based governance. Linking such systems to participatory monitoring enhances transparency and policy responsiveness.

Together, these clusters demonstrate that health equity can only be advanced through multi-sectoral, anticipatory, and inclusive strategies that align with SDH, Human Capital, and Life-Course frameworks.

### Limitations and future research directions

This review provides a systematic synthesis of evidence on how ageing, migration, and socioeconomic disparities intersect to shape health equity in the post-pandemic era. Three limitations must be acknowledged.

First, despite rigorous search strategies, the evidence base is affected by publication bias, with studies disproportionately drawn from high-income countries. Grey literature from the Global South remains scattered or difficult to access. To mitigate this, institutional reports (WHO, UN, IOM) and national policy documents were integrated alongside peer-reviewed sources to improve geographic and institutional balance.

Second, the inclusion of both academic and institutional literature introduced heterogeneity in methodological quality. To address this, the Critical Appraisal Skills Programme (CASP) checklist was applied to peer-reviewed studies, while an adapted Joanna Briggs Institute (JBI) framework was used for grey literature, ensuring comparability and analytical rigour.

Third, the synthesis relied on interpretive thematic coding, which may carry subjectivity. Credibility was enhanced through iterative coding, peer validation, triangulation across data sources, and transparent memo-writing of analytical decisions.

Future research should prioritise underrepresented contexts, particularly fragile states, rapidly urbanising regions, and countries in Latin America, the Pacific, and Eastern Europe. Longitudinal and ethnographic studies can illuminate how intersecting vulnerabilities across age, gender, migration status, and informal work trajectories accumulate over time. In addition, participatory approaches that engage older adults and mobile populations are vital to bridge the gap between policy intent and lived realities. Finally, emerging themes such as digital exclusion as a social determinant of health and intergenerational transfers under crisis conditions deserve deeper empirical exploration.

## Conclusion

The COVID-19 pandemic magnified structural inequities at the nexus of ageing, migration, and socioeconomic disparity. This review demonstrates that achieving health equity requires more than biomedical reform: it demands an intersectional policy agenda grounded in the Social Determinants of Health, Human Capital, and Life-Course frameworks.

Three policy priorities emerge. First, strengthen health systems by embedding geriatric and migrant-sensitive care into universal coverage. Ghana’s National Health Insurance Scheme exemptions and South Africa’s ICOPE programme illustrate scalable models. Second, expand social protection through inclusive pensions and labour protections that reach informal sector workers and older women, as seen in South Africa’s Old Age Grant and India’s Unorganised Workers’ Social Security Act. Third, improve migration governance and data systems by integrating ageing into migration policy, expanding age-disaggregated data, and fostering regional cooperation, building on the AU Agenda 2063, the IOM Migration Health Strategy, and the UN Decade of Healthy Ageing.

Collectively, these reforms demand coordinated governance across health, labour, and social protection sectors, supported by robust monitoring frameworks. Transforming the rhetoric of equity into measurable outcomes will require courageous political choices, disaggregated data, and participatory governance that centres the voices of older adults and mobile populations.

Health equity in the 21st century is both a development imperative and a moral, intergenerational obligation.

## Data Availability

All data used in this review are derived from published sources. The full list of included studies and quality appraisal results is available upon reasonable request.
